# Supplementary material for: The protective association between statins use and adverse outcomes among COVID-19 patients: A systematic review and meta-analysis
Source: PLoS One. 2021 Jun 24;16(6):e0253576. doi: 10.1371/journal.pone.0253576 (PMC8224908; doi:10.1371/journal.pone.0253576)
Supplement: S1 Appendix — (DOCX) [file pone.0253576.s001.docx]

**S1 Appendix. Search strategies.**

Database: Ovid MEDLINE(R) and Epub Ahead of Print, In-Process & Other Non-Indexed Citations, Daily and Versions(R) <1946 to December 04, 2020> Search Strategy:

--------------------------------------------------------------------------------

1 (Covid-19 or Covid19).mp. (75787)

2 SARS-CoV-2.mp. (24835)

3 severe acute respiratory syndrome coronavirus 2.mp. (37456)

4 or/1-3 (78351)

5 exp Hydroxymethylglutaryl-CoA Reductase Inhibitors/ (41254)

6 statin*.mp. (44888)

7 exp Atorvastatin/ (6644)

8 (Atorvastatin or Lipitor).mp. (9966)

9 (Cerivastatin or Baycol).mp. (784)

10 exp Fluvastatin/ (1398)

11 (Fluvastatin or Lescol).mp. (2109)

12 exp Lovastatin/ (11204)

13 (Lovastatin or Mevacor or Altoprev).mp. (5988)

14 exp Pravastatin/ (3434)

15 (Pravastatin or Lipostat).mp. (4941)

16 exp Rosuvastatin Calcium/ (2530)

17 (Rosuvastatin or Crestor).mp. (3958)

18 exp Simvastatin/ (7807)

19 (Simvastatin or Zocor).mp. (11151)

20 (Pitavastatin or Livalo).mp. (1001)

21 or/5-20 (67164)

22 4 and 21 (162)

23 limit 22 to (english language and yr="2019 -Current") (159)

***************************

Database: Embase Classic+Embase <1947 to 2020 Week 49> Search Strategy:

--------------------------------------------------------------------------------

1 (Covid-19 or Covid19).mp. (67997)

2 SARS-CoV-2.mp. (23756)

3 severe acute respiratory syndrome coronavirus 2.mp. (22209)

4 or/1-3 (74109)

5 exp hydroxymethylglutaryl coenzyme A reductase inhibitor/ (159145)

6 statin*.mp. (78372)

7 exp atorvastatin/ (38132)

8 (Atorvastatin or Lipitor).mp. (38991)

9 exp cerivastatin/ (3864)

10 (Cerivastatin or Baycol).mp. (3922)

11 exp fluindostatin/ (9543)

12 (Fluvastatin or Lescol).mp. (3326)

13 exp mevinolin/ (16014)

14 (Lovastatin or Mevacor or Altoprev).mp. (5973)

15 exp pravastatin/ (20066)

16 (Pravastatin or Lipostat).mp. (20522)

17 exp rosuvastatin/ (15501)

18 (Rosuvastatin or Crestor).mp. (15827)

19 exp simvastatin/ (38189)

20 (Simvastatin or Zocor).mp. (39664)

21 exp pitavastatin/ (3255)

22 (Pitavastatin or Livalo).mp. (3341)

23 or/5-22 (180346)

24 4 and 23 (315)

25 limit 24 to (english language and yr="2019 -Current") (312)

***************************

Database: EBM Reviews - Cochrane Central Register of Controlled Trials <November 2020>

Search Strategy:

--------------------------------------------------------------------------------

1 (Covid-19 or Covid19).mp. (3412)

2 SARS-CoV-2.mp. (1278)

3 severe acute respiratory syndrome coronavirus 2.mp. (246)

4 or/1-3 (3473)

5 exp Hydroxymethylglutaryl-CoA Reductase Inhibitors/ or hydroxymethylglutaryl coenzyme A reductase inhibitor*.mp. (7316)

6 statin*.mp. (10414)

7 exp Atorvastatin/ (0)

8 (Atorvastatin or Lipitor).mp. (5556)

9 (Cerivastatin or Baycol).mp. (172)

10 exp Fluvastatin/ or fluindostatin.mp. (171)

11 (Fluvastatin or Lescol).mp. (722)

12 exp Lovastatin/ or mevinolin.mp. (2263)

13 (Lovastatin or Mevacor or Altoprev).mp. (936)

14 exp Pravastatin/ (1012)

15 (Pravastatin or Lipostat).mp. (2012)

16 exp Rosuvastatin Calcium/ (0)

17 (Rosuvastatin or Crestor).mp. (2605)

18 exp Simvastatin/ (1783)

19 (Simvastatin or Zocor).mp. (3964)

20 (Pitavastatin or Livalo).mp. (532)

21 or/5-20 (19081)

22 4 and 21 (25)

23 limit 22 to (english language and yr="2019 -Current") (3)

***************************
