# Supplementary material for: The protective association between statins use and adverse outcomes among COVID-19 patients: A systematic review and meta-analysis
Source: PLoS One. 2021 Jun 24;16(6):e0253576. doi: 10.1371/journal.pone.0253576 (PMC8224908; doi:10.1371/journal.pone.0253576)
Supplement: S2 Appendix — (DOCX) [file pone.0253576.s002.docx]

**S2 Appendix. PRISMA flow diagram.**

21 studies identified for potential quantitative synthesis

297 records excluded

49 full-text articles assessed for eligibility

346 records screened after duplicates removed (n=136)

474 records identified through database search

8 additional records identified

28 full-text articles excluded:

- Commentary article (n = 18)
- Review article (n = 4)
- Study protocol (n = 2)
- Does not report results by statin use (n = 4)

12 studies reporting on 13 cohorts included in quantitative synthesis (meta-analysis)

13 studies reporting on 14 cohorts included in systematic review
