# Supplementary material for: The protective association between statins use and adverse outcomes among COVID-19 patients: A systematic review and meta-analysis
Source: PLoS One. 2021 Jun 24;16(6):e0253576. doi: 10.1371/journal.pone.0253576 (PMC8224908; doi:10.1371/journal.pone.0253576)
Supplement: S3 Appendix — (DOCX) [file pone.0253576.s003.docx]

**S3 Appendix. Assessment for publication bias – funnel plot.**
